# Study of Properties of Novel Geopolymers Prepared with Slate Stone Cutting Sludge and Activated with Olive Stone Bottom Ash

**DOI:** 10.3390/ma18081774

**Published:** 2025-04-13

**Authors:** Elena Picazo Camilo, Juan José Valenzuela Expósito, Raúl Carrillo Beltrán, Griselda Elisabeth Perea Toledo, Francisco Antonio Corpas Iglesias

**Affiliations:** Higher Polytechnic School of Linares, University of Jaén, 23700 Linares, Jaén, Spain; jjexposi@ujaen.es (J.J.V.E.); rcb00024@red.ujaen.es (R.C.B.); gept0001@red.ujaen.es (G.E.P.T.); facorpas@ujaen.es (F.A.C.I.)

**Keywords:** geopolymers, mining waste, bottom biomass ash, chamotte, sustainable materials

## Abstract

The sustainable development of building materials is based on reusing by-products to reduce environmental impact and promote alternatives to traditional materials. In this study, geopolymers were developed from by-products of the mining, ceramic, and thermal industries: slate stone cutting sludge (SSCS) and chamotte (CH) as aluminosilicate sources, and olive stone bottom ash (OSBA) as an alkaline activator, combined with sodium silicate (Na_2_SiO_3_). Eight geopolymer families were prepared with constant amounts of SSCS and CH and varying proportions of OSBA/Na_2_SiO_3_ (0.88–1.31). The evaluation phase included physical, chemical, mechanical, and microstructural tests. The results showed that the optimum geopolymer formulation (GP E) contained 25% SSCS, 15% CH, and 19% OSBA with a Na_2_SiO_3_/OSBA ratio of 1.0, achieving a compressive strength of 24.12 MPa after 28 days of curing. GP E also showed the lowest porosity (19.54%), minimal water absorption (6.86%), and favorable thermal conductivity (0.688 W/mK). Fourier transform infrared spectroscopy (FTIR) and scanning electron microscopy (SEM) confirmed the formation of dense and homogeneous matrices. These results demonstrate the feasibility of manufacturing geopolymers using SSCS, CH, and OSBA as substitutes for traditional binders, promoting sustainable practices, reusing industrial by-products, and reducing carbon emissions in construction.

## 1. Introduction

Global energy consumption for air conditioning of buildings represents a significant proportion of total energy consumption. This is largely due to poor thermal insulation systems that increase the demand for heating and cooling. As a consequence, carbon dioxide (CO_2_) emissions from the generation of this energy contribute substantially to climate change [1,2]. According to estimates by the International Energy Agency (IEA), 34,116 Mt of CO_2_ were emitted from burning fuels to meet building energy demand, which constitutes 28% of global energy [3,4,5], and much of this is wasted through avoidable heat losses associated with poor insulation. This highlights the urgent need to develop innovative materials that improve the thermal performance of buildings and contribute to the reduction in greenhouse gas emissions.

Ceramic tiles are widely used in roof construction due to their mechanical, insulating, and weathering properties. However, their manufacturing process requires high amounts of energy and raw materials. Conventional production involves firing clays at temperatures above 1000 °C, a process that requires large amounts of energy and generates approximately 0.6 tons of CO_2_ per ton of material produced [6,7].

In addition, the extraction of raw materials such as clays has a considerable environmental impact, as it contributes to soil degradation and loss of biodiversity in the extraction areas. In terms of greenhouse gas emissions, traditional ceramic tiles represent a significant source due to the use of fossil fuels in the manufacturing stages [8]. These characteristics highlight the need to research and develop alternatives that reduce dependence on virgin raw materials and employ less polluting manufacturing processes.

Roofs play a key role in the energy efficiency of buildings, as they are pathways for heat loss or gain. Insulating materials used in roofs, such as ceramic tiles, should provide protection against inclement weather and contribute to minimizing energy consumption by improving thermal insulation. In this context, numerous studies have focused on the application of various materials and technologies to improve the thermal properties of roofs. These include the thermal insulation of roofs coated with fibers and resins, tire rubber, double-layer systems composed of glass wool and aerogel, or combinations of sandwich panels with insulating materials such as rigid foams and lightweight roofing panels [9,10,11,12]. Although these solutions have improved thermal performance, many of them increase the cost of roofing systems and require more complex manufacturing processes.

The search for more sustainable and efficient alternatives has led to exploring the integration of industrial waste and by-products in the manufacture of roofing materials. These approaches enhance the insulating properties of the final material and contribute to reducing the environmental impact associated with its production. Developments in this field seek solutions that combine sustainability, energy efficiency, and economic viability, a critical balance to promote their adoption in the construction market.

Geopolymers have emerged as an innovative and sustainable solution for the construction industry. These materials are obtained by the alkaline activation of aluminosilicate-rich precursors, such as industrial waste (aluminum powder, fly ash, blast furnace slag, and construction and demolition waste) and various industrial wastes [13,14,15,16,17]. This chemical process produces three-dimensional structures similar to traditional mortars, but with a significantly lower carbon footprint due to the low curing temperatures required and the revaluation of waste.

One of the main advantages of geopolymers is their ability to integrate waste as raw materials, promoting the circular economy. In addition, their versatility allows them to be adapted to various applications in construction, from coatings to structural elements. The search for alternative precursors, especially those from waste, is an active field of research that not only contributes to the sustainability of the sector but also offers a viable solution to the problems associated with industrial waste management.

**Figure 1:**
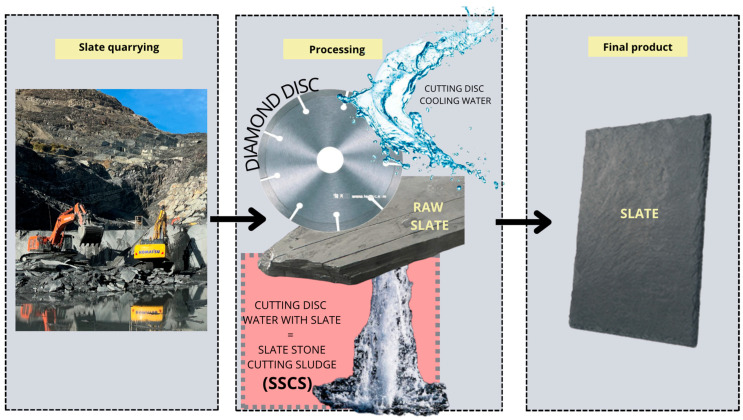
Slate extraction and production process.

The mining industry generates a large amount of waste associated with its extraction and processing process, such as slate stone cutting sludge (SSCS), which often accumulates in landfills, generating negative impacts on the environment [18]. Among these, slate waste stands out for its potential as a precursor material in the manufacture of geopolymers due to its richness in silicates and aluminosilicates [19,20,21]. In regions where slate mining is predominant, such as northern Spain, these wastes represent a source that could be valorized for construction applications. Their use would reduce the environmental impact associated with their deposition and contribute to the creation of high-value-added products, promoting more sustainable practices in the mining industry. Figure 1 shows the slate production process where SSCS is generated as waste from the slate industry.

Biomass bottom ashes (BBA), generated as a residue in combustion processes for energy generation, have emerged as a promising alternative in the synthesis of geopolymeric materials because of their use as a sustainable alkaline activator. These ashes contain reactive compounds such as calcium, potassium, and sodium oxides that can act as alkaline activators, partially or completely replacing commercial activators such as sodium hydroxide (NaOH) or sodium silicate (Na_2_SiO_3_). The use of ashes as a partial substitute for commercial alkaline activators has been extensively studied with ashes from various industries including those from coal-fired power plants [22,23,24] or waste incineration plants [22,25,26], construction by-products [27,28], or from the mining sector [29,30]. In the field of ash applications, biomass fly ash (BFA) stands out due to its smaller particle size, which increases its reactivity [31,32,33]. However, several studies focus on the use of BBA [34,35,36] due to the significant quantities generated from this type of waste, estimated at 10 million tons per year [37].

In the field of geopolymeric materials, biomass ashes have been shown to be effective in improving the mechanical and chemical properties of the final product. These ashes, especially those derived from the combustion of silica- and calcium-rich residues, such as olive stone bottom ash (OSBA), act as activators by providing reactive components that enrich the geopolymer matrix. Recent studies have shown that the incorporation of OSBA can improve compressive strength, decrease porosity, and contribute to the development of denser and more durable structures [38,39,40,41].

**Figure 2:**
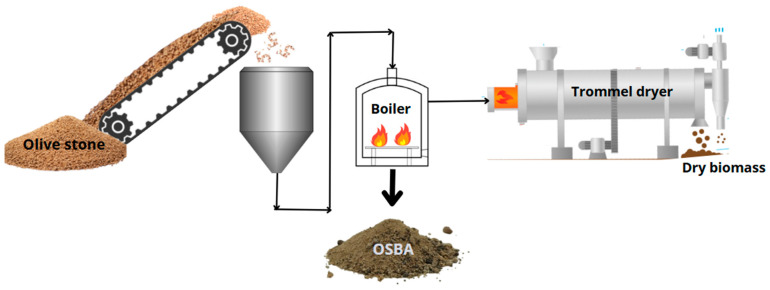
Generation of OSBA in a thermal power plant.

Regions such as Andalusia (Spain), with a high production of biomass derived from the olive industry, generate large quantities of OSBA from the cogeneration of olive oil production by-products (Figure 2), which represents a strategic opportunity for the valorization of this waste. The integration of OSBA in geopolymers fosters a circular economy approach and offers innovative solutions for sustainable construction, aligned with the Sustainable Development Goals (SDGs).

The geopolymerization process using mining waste as a source of aluminosilicates and OSBA as a sustainable alkaline activator is an alternative for the development of in situ materials. This technology offers a viable solution for the management of mining and industrial waste while providing quality materials with thermal and durable properties. The application of geopolymers has the potential to revolutionize the construction industry, promoting more sustainable practices and significantly reducing the sector’s environmental impact.

## 2. Materials and Methods

### 2.1. Raw Materials and Properties

Slate stone cutting sludge (SSCS), used as a precursor material for geopolymers, is obtained from the processing of slate by diamond blade cutting from the Matacouta mining activity (La Cabrera Baja (León, Spain)) and consists of a mixture of water and slate dust. SSCS was combined with CH supplied by Cerámica San Francisco (Bailén (Jaén, Spain)) as a source of aluminosilicates. The alkaline activator used was developed from varying proportions of Na_2_SiO_3_ (29.2% SiO_2_, 8.9% Na_2_SO_3_, and 61.9% H_2_O) (Panreac) and OSBA supplied by Garzón Green Energy (Bailén (Jaén, Spain)) from a biomass boiler for the production of thermal energy from olive pits.

Elemental analysis of SSCS, CH, and OSBA in Truspec Micro (Table 1) showed that the carbonate content was low, indicating the suitability of the materials for use in the geopolymerization reaction. High carbonate contents are related to low alkalinity, which results in the precursor raw materials not dissolving completely, forming partial networks and affecting the final mechanical and chemical properties of the material [42].

**Figure 3:**
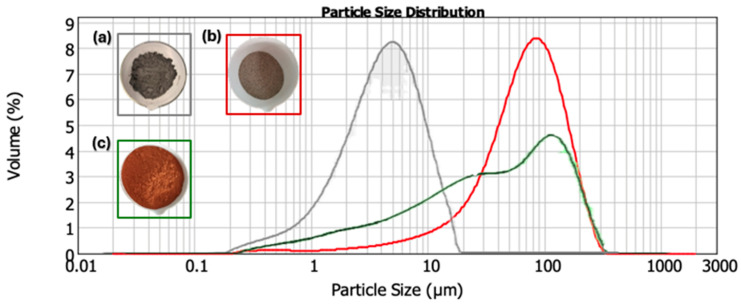
Granulometric distribution of (**a**) SSCS, (**b**) OSBA, and (**c**) CH.

The particle size distribution of SSCS, CH, and OSBA was determined using the Malvern Mastersizer 2000 (Figure 3). The particle size influences the geopolymerization reaction by favoring better packing of the grains [43]. The particle size of SSCS is directly influenced by the machining process and was found to have a mean particle size (D_50_) of 5.179 µm and a specific area of 1.88 m^2^/g. OSBA used in the experimental development were pre-treated by calcination at 950 °C to increase reactivity [44], ground in a Retsch PM200 planetary mill, and sieved to a particle size of less than 300 µm (D_50_ of 70.618 µm and specific area of 0.321 m^2^/g). Similarly, CH was subjected to the same physical pre-treatment (grinding and sieving) with 90% of its grains (D_90_) smaller than 300 µm (239.542 µm) and a specific area of 0.794 m^2^/g.

The reactivity of the particles is related to several factors such as crystallinity, chemical composition of the precursors and alkali activators, and the density (Table 2) and morphology of the particles [45]. The density of the materials composing geopolymer influences their specific surface areas. Lower densities lead to higher specific surface areas due to less compaction of the grains, which facilitates the dissolution of the particles in the alkaline medium and provides higher availability of Si^4+^ and Al^3+^ ions needed for gel formation. Consequently, higher densities provide smaller contact areas and therefore tend to be less reactive with the alkaline activator, which slows down the dissolution of aluminosilicates [46].

**Figure 4:**
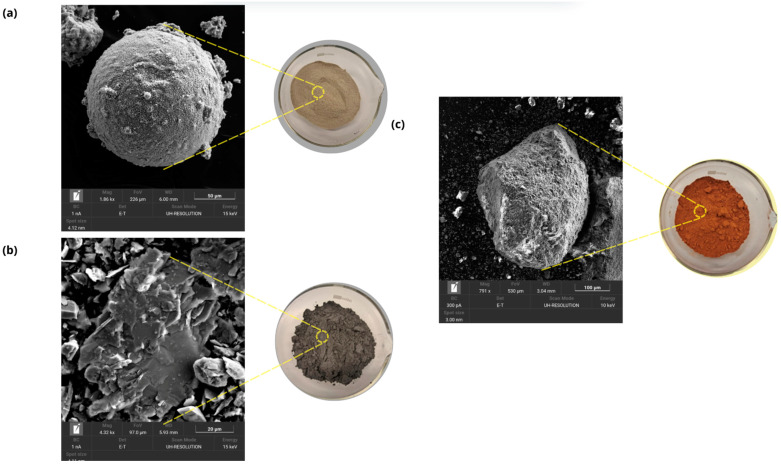
Physical appearance and SEM of: (**a**) OSBA, (**b**) SSCS, and (**c**) CH.

The particle morphology (Figure 4) determined by Carl Zeiss Merlin Scanning Electron Microscope (SEM, Zeiss GmbH, Jena, Germany) shows the spherical morphology of OSBA. SSCS shows different exfoliation planes typical of shales [47], while CH shows a rough and porous texture typical of clay [48].

The chemical composition of the raw materials, determined by X-ray fluorescence (XRF, Bruker AXS GmbH, Karlsruhe, Germany) on Bruker’s Pioner S4 Explorer, showed that SSCS and CH were suitable precursors for the geopolymerization reaction due to their high Al_2_O_3_ (21. 97 and 15.41%, respectively) and SiO_2_ (50.34 and 58.98%, respectively) with SiO_2_/Al_2_O_3_ ratios of 2.29 and 3.82, which are within the recommended range of 1 to 5 to promote alkali gel formation [49]. OSBA shows a high content of K_2_O (27.48%) and CaO (34.22%), which favors its use as an alkaline activator and the formation of the gel composed of Si-O-Si and Si-O-Al bonds as a precursor to the formation of the solid matrix that subsequently reorganizes and condenses into a three-dimensional structure [50,51,52]. Table 3 shows the chemical composition of SSCS, OSBA, and CH.

**Figure 5:**
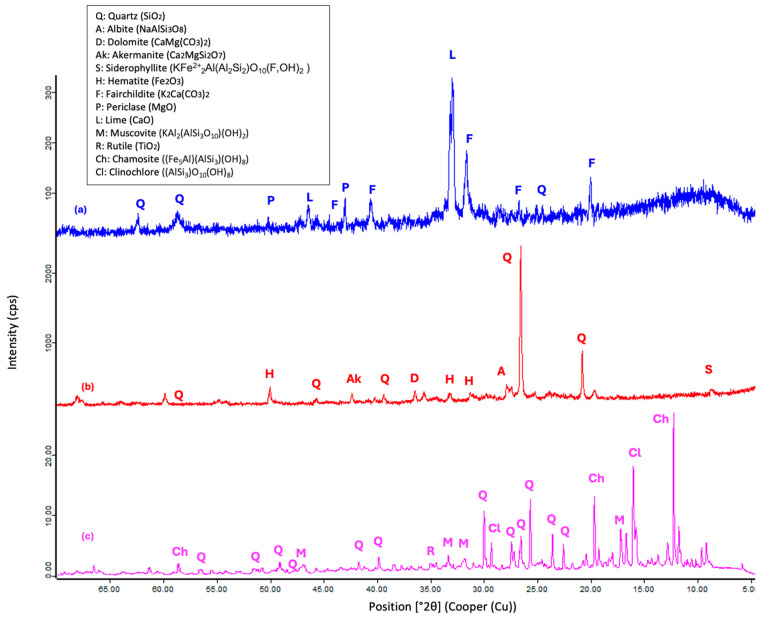
XRF patterns: (**a**) OSBA, (**b**) CH, and (**c**) SSCS.

The X-ray diffraction (XRD) patterns of SSCS, OSBA, and CH obtained using PANalytical’s X’Pert Pro (Malvern Analytical Ltd., Malvern, UK) (Figure 5) were measured from 4° to 70° (2θ) with a step size of 0. 0167° and show that the mineralogical composition of SSCS is composed of predominant oxides (SiO_2_ and Al_2_O_3_) appearing as quartz (Q) (SiO_2_), muscovite (M) (KAl_2_(AlSi_3_O_10_)(OH)_2_), and K_2_O. Fe_2_O_3_ and MgO are present as clinochlore (Cl) ((AlSi_3_)O_10_(OH)_8_) and chamosite (Ch) ((Fe_5_Al)(AlSi_3_)(OH)_8_) and TiO_2_ appears as rutile (R). OSBA shows fairchildite (F) (K_2_Ca(CO_3_)_2_) and siltstone (L) (CaO) in the form of K_2_O and CaO, respectively, and traces of quartz (Q) (SiO_2_) and periclase (P) (MgO). The mineralogical phases of CH highlight crystalline phases with the presence of SiO_2_ and Al_2_O_3_ in the form of quartz (Q) (SiO_2_) as in SSCS. Fe_2_O_3_ appears as haematite (H) and CaO as dolomite (D) (CaMg(CO_3_)_2_) and akermanite (Ak) (Ca_2_MgSiO_7_).

**Figure 6:**
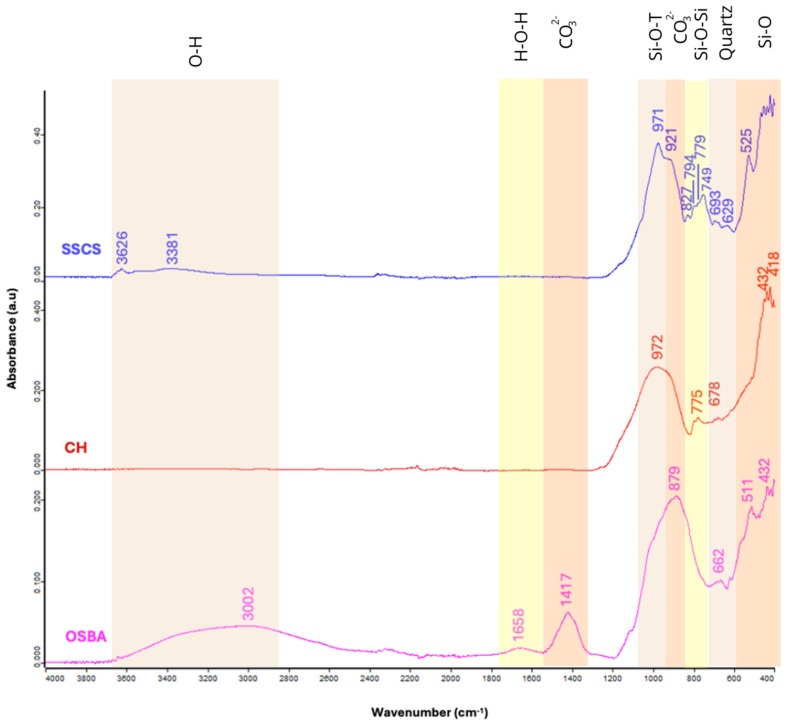
FTIR patterns: SSCS, CH, and OSBA.

The identification of the functional groups of SSCS, OSBA, and CH (Figure 6) was carried out by Fourier transform infrared spectroscopy (FTIR) on a Jasco Analytica model 6800 FV (Jasco International Co., Ltd., Heckmondwike, UK). The identification range was between 4000 and 400 cm^−1^.

SSCS and OSBA show bands appearing in the range 3626–3002 cm^−1^ related to the O-H stretching vibration, indicating the presence of adsorbed water [53]. In addition, at 1648 cm^−1^, a peak appears in OSBA related to the H-O-H bending vibration representing crystalline water in hydration products [53,54] and another at 1417 cm^−1^ associated with the asymmetric C-O stretching vibration related to carbonate phases [55]. The bands located between 972 and 921 cm^−1^ in SSCS and CH indicate an asymmetric Si-O-T stretching vibration common in silica-rich materials (50.34 and 58.98%, respectively) as indicated by compositional analysis (Table 3) [56,57]. At 879 and 827 cm^−1^, vibrations of CO bonds in carbonate groups are detected in OSBA and SSCS, respectively, which are related to the formation of carbonates by the exposure of the material to atmospheric CO_2_ or by-products present [58,59]. The peaks characteristic of Si-O-Si symmetric stretching bending appear between 800 and 700 cm^−1^ in SSCS and CH [58,59,60]. The vibration bands between 700 and 600 cm^−1^ are related to the presence of quartz in SSCS, OSBA, and CH, whose presence is verified in the diffractogram (Figure 5) [60]. The vibrational bands between 525 and 418 cm^−1^ correspond to Si-O bending vibrations [61,62]. Table 4 shows the characteristic bands for each wavenumber of SSCS, OSBA, and CH.

### 2.2. Mixture Design and Methodology

Based on preliminary experiments, the development of 1 control family (GP A) composed of commercial alkaline activators (Na_2_SiO_3_ and NaOH (98% NaOH, 1% Na_2_SiO_3_, traces)) and 7 families developed with the sustainable alkaline activator (OSBA and Na_2_SiO_3_) was determined. The selection of NaOH as the alkaline activator was influenced by the workability of the mixtures observed in previous work and the higher activation efficiency compared to potassium hydroxide (KOH) due to the smaller Na^+^ size [63,64]. Therefore, 8 families with different substitution ratios of Na_2_SiO_3_ (28, 29, 30, 31, 32, 33, 34, and 37%), OSBA (0, 15, 16, 17, 18, 19, 20, and 21%), and NaOH (0 and 17%) were experimentally developed. The proportions of SSCS (25%) and CH (15%) were kept constant. The amount of distilled water (W_d_) increased for increasing amounts of OSBA to improve the workability of the geopolymer gel due to the higher absorption related to particle porosity and higher surface area increasing the water demand [65]. The liquid/binder ratio in the control family (GP A) was 1.86, while in the families developed with OSBA and Na_2_SiO_3_ the ratio was kept constant at 1.50. Table 5 shows the ratios of the 8 developed families and the pH of the alkaline solutions and Table 6 shows the Si/Al, K/Si, Na/Si, and Ca/Si molar ratios of the geopolymer families.

**Figure 7:**
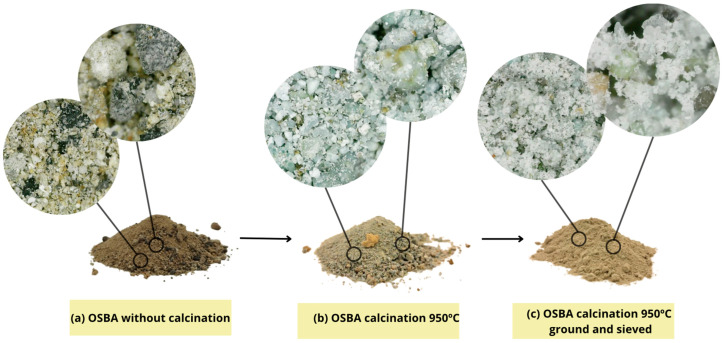
OSBA process: (**a**) without calcination, (**b**) calcination 950 °C, and (**c**) calcination 950 °C, ground and sieved.

The sustainable alkaline activator based on OSBA was prepared with the ash previously calcined at 950 °C to increase the reactive species of silicon and aluminum [51] for 1 h and then ground and sieved at 300 µm to reduce the particle size, decrease the specific surface area, and improve the interaction with Na_2_SiO_3_ [66] (Figure 7). OSBA was diluted in W_d_ and mixed on the Nahita model 690-1 magnetic stirrer for 10 min. Na_2_SiO_3_ was then added and stirring was continued for a further 5 min. The pH of the alkaline activators was monitored using a Hanna Edge pH meter and a pH above 12 was taken as a reference value to promote the geopolymerization reaction [67]. The procedure followed with the commercial alkaline activator is identical, where NaOH pellets were dissolved in W_d_ for 10 min, after which Na_2_SiO_3_ was added and stirred for a further 5 min.

**Figure 8:**
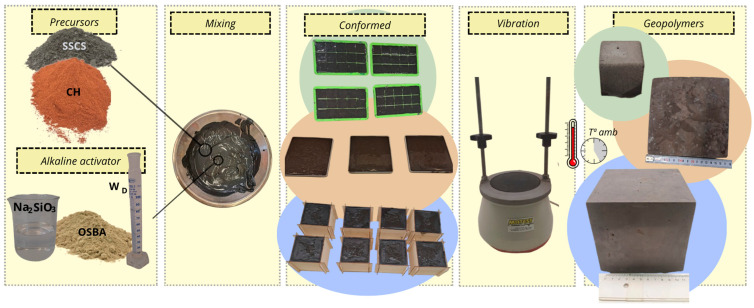
Geopolymer manufacturing process.

The geopolymer binder was prepared by first mixing the SSCS and CH precursors in the Proeti planetary mixer for 5 min. Then, the previously prepared alkaline solution was added and mixed for 10 min until a homogeneous mass was obtained. The specimens were formed by pouring the material into silicone molds and vibrating on a vibrating table to eliminate the air contained in the mixture and to avoid large pores. Molds of different dimensions were used to adapt the specimens to the standards of the tests carried out. Six 3.5 × 3.5 × 3.5 cm specimens, one 10 × 10 × 10 × 10 × 10 cm specimen, and three 18 × 18 × 4 cm specimens were made from each of the geopolymer families. All the specimens were cured at a controlled ambient temperature of 21 ± 5 °C for 4 days in the mold and 24 days without mold. Figure 8 shows the scheme of the process followed for the manufacture of the geopolymers.

The conformed geopolymers were tested in order to evaluate their properties. All the tests were performed under their corresponding standards, which are specified in Table 7.

**Figure 9:**
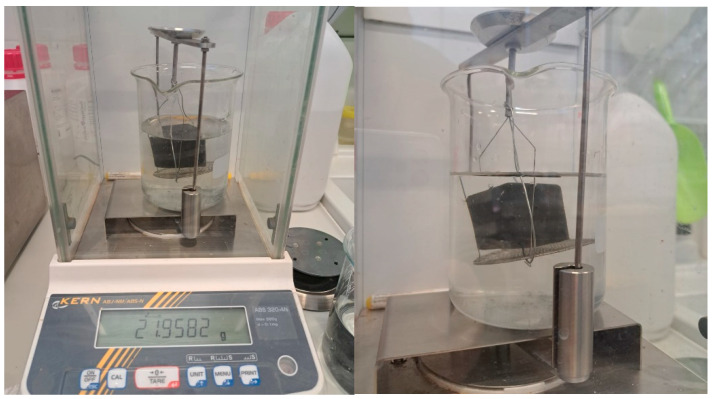
Determination of porosity and bulk density in hydrostatic balance.

The physical characterization of the geopolymers was carried out on 6 specimens per family of dimensions 3.5 × 3.5 × 3.5 cm. The mass variation in the specimens is related to the linear shrinkage caused by capillary water loss during the curing process [76]. The mass loss of the specimens was determined by the weighed mass difference, while the dimensional variation was determined by the dimensional difference before and after the curing process at the set conditions. Water transport by capillary action and immersion were assessed by weighing on a Cobos RB-30KG (Balanzas Cobos, Hospitalet de Llobregat, Spain) balance accurate to 0.001 g and the weight after the elapsed time in the thermostatic bath stipulated in the standards quoted in Table 7. The importance of capillary absorption lies in the evaluation of the behavior of the material in outdoor environments or in intermittent contact with water, while immersion absorption is based on the evaluation of the maximum degree of saturation of the material. Both are influenced by pore size related to the degree of particle compactness, open porosity, and bulk density [77]. The open porosity and bulk density were determined on a hydrostatic balance by determining the submerged mass and the saturated mass (Figure 9).

**Figure 10:**
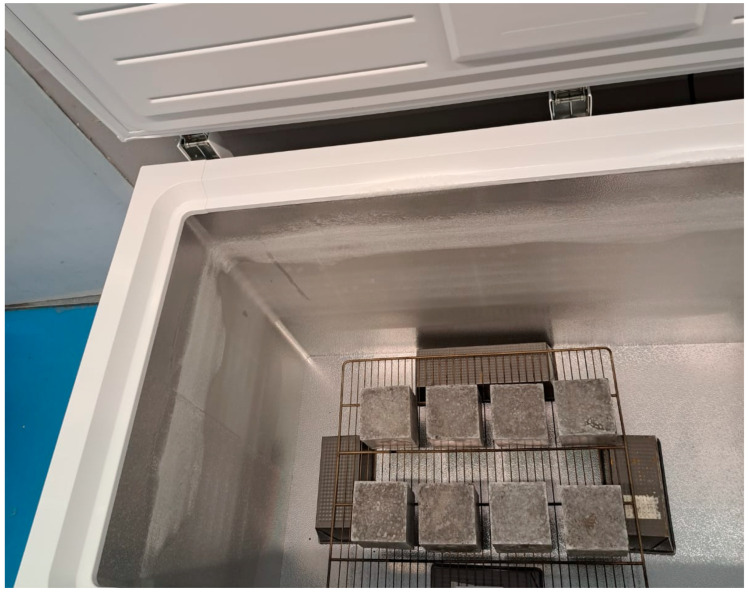
Geopolymers during the freezing period for the freeze–thaw test.

To evaluate the resistance and durability of the geopolymers to extreme climatic conditions, the resistance to mechanical damage was evaluated by freeze–thaw cycles on a test specimen of dimensions 10 × 10 × 10 cm for each of the families (Figure 10). The test was carried out in a freezer by placing the specimens on a grid and keeping them at a temperature of −15 ± 2 °C for 8 hours. After the freezing period, the specimens were kept for 8 h in a controlled atmosphere at 20 ± 2 °C and 95% relative humidity. Fifteen freeze–thaw cycles were carried out. In this way, the stability of the microstructure was tested because the open porosity is more susceptible to damage by water penetration and expansion during freezing, which can cause cracks in the material [78].

**Figure 11:**
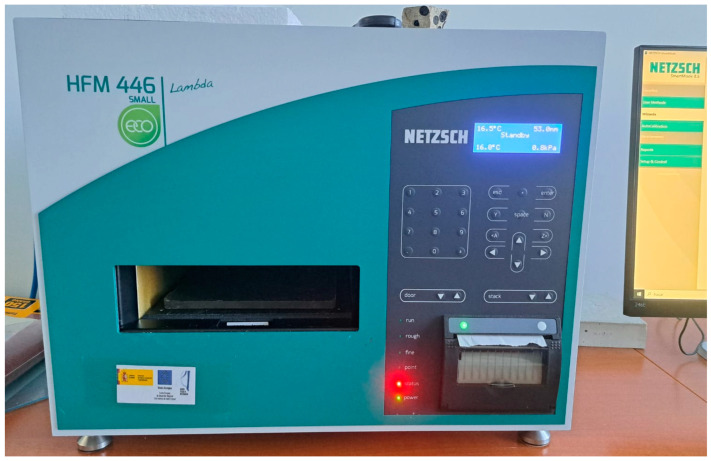
Determination of thermal conductivity in Netzsh HFM 446 Lambda.

To measure the thermal conductivity, 3 specimens of dimensions 20 × 20 × 4 cm were molded for each of the families. Each of the specimens was introduced into the HFM 446 Lambda Eco-Line Netzsch (Netzsch, High Franconia, Germany) (Figure 11) consisting of dual heat flux transducers, which were previously calibrated with reference materials with known conductivity.

**Figure 12:**
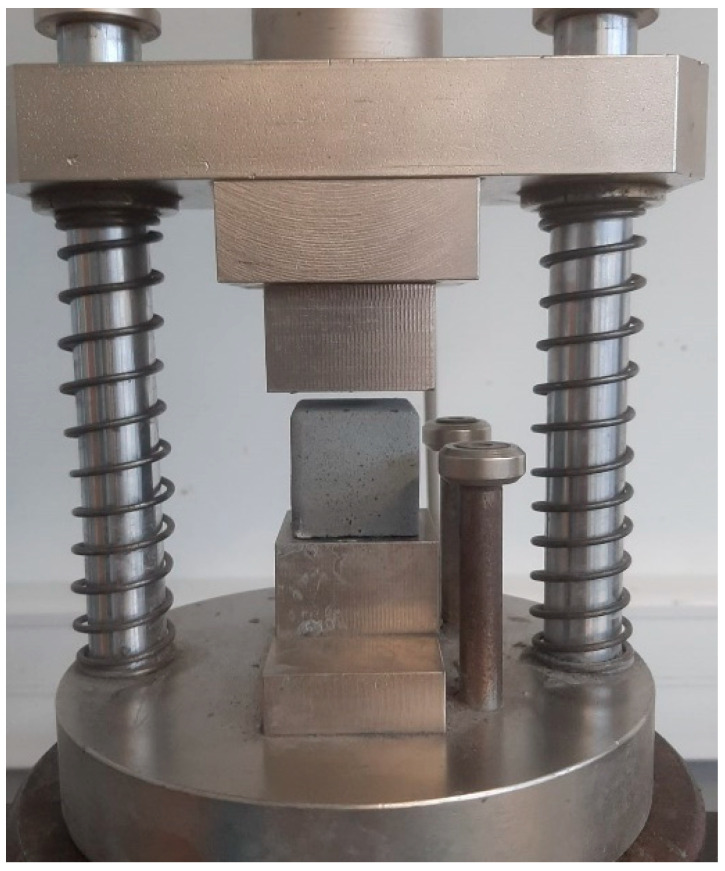
Determination of compressive strength in Shimadzu AG-300 KNX.

The determination of the compressive strength was carried out by testing the 35 × 35 × 35 cm specimens in the Shimadzu AG-300 KNX equipment (Shimadzu, Korneuburg, Austria). For this purpose, the specimens were subjected to rupture at 7, 14, and 28 days of curing (Figure 12). Finally, the geopolymers were analyzed by FTIR to verify the effective incorporation of the precursors into the matrix and to identify the formation of new chemical phases resulting from the alkaline activation process. In addition, the specimens were examined by scanning electron microscopy (SEM) to evaluate the microstructural properties by observing particle distribution, porosity, homogeneity of the geopolymer matrix, and the presence of unreacted phases.

The microstructural chemical characterization of the specimens was carried out by XRD (Pioner S4 Explorer Bruker, Bruker AXS GmbH, Karlsruhe, Germany) and FTIR (FT-IR Vertex 70 Bruker, Bruker AXS GmbH, Karlsruhe, Germany) analyzing the 8 families formed. The XRD test was performed at a 2θ scanning angle between 4° and 70° with a step size of 0.0167°. The tube was operated at 45 kV and 40 mA and the sample was rotated at 10 rpm during the measurement.

## 3. Results and Discussions

### 3.1. Physical Characterization of Geopolymer

Table 8 shows the results obtained from the physical characterization of the 8 families formed after 28 days of curing at ambient temperature (21 ± 5 °C).

#### 3.1.1. Determination of Mass Loss

**Figure 13:**
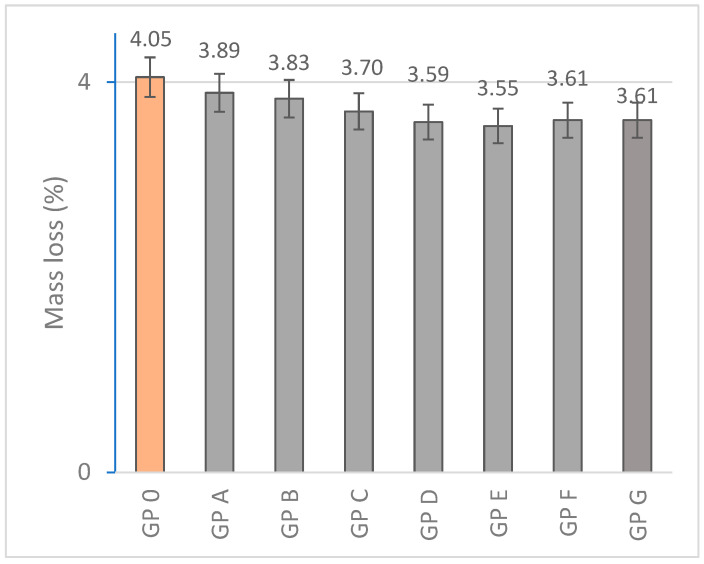
Mass loss (%) of geopolymers.

The results obtained for mass loss (Figure 13) show values close to each other after 28 days of curing for all the formed families; however, a decreasing trend is observed with the increasing OSBA content. The amount of water incorporated into the mixtures is controlled to ensure the mobility of the reagents and to facilitate the geopolymerization reaction. Mass losses are related to the evaporation of free water that does not participate in the reaction during the curing process. The control family (GP 0) showed a slightly higher mass loss percentage (4.05%) than those experienced by the OSBA- and Na_2_SiO_3_-activated geopolymer families (between 3.89 and 3.55%). NaOH in combination with Na_2_SiO_3_ are highly reactive alkaline activators that favor a rapid dissolution of the precursor particles. This results in a higher amount of water being generated in the formation of the geopolymer gel, with some of it remaining as free water trapped in the matrix as it is not fully incorporated into the gel structure. During the curing process, the free water evaporates, contributing to the higher mass loss observed in GP 0. On the other hand, as the OSBA content increases, there is a decrease in mass loss associated with the increase in specific surface area, which influences a higher efficiency in the chemical reaction by increasing the formation of the geopolymer gel and a higher physical interaction with water as the water is trapped in the pores or on the surface of the OSBA particles, decreasing the amount of water lost during curing.

#### 3.1.2. Determination of Linear Shrinkage

**Figure 14:**
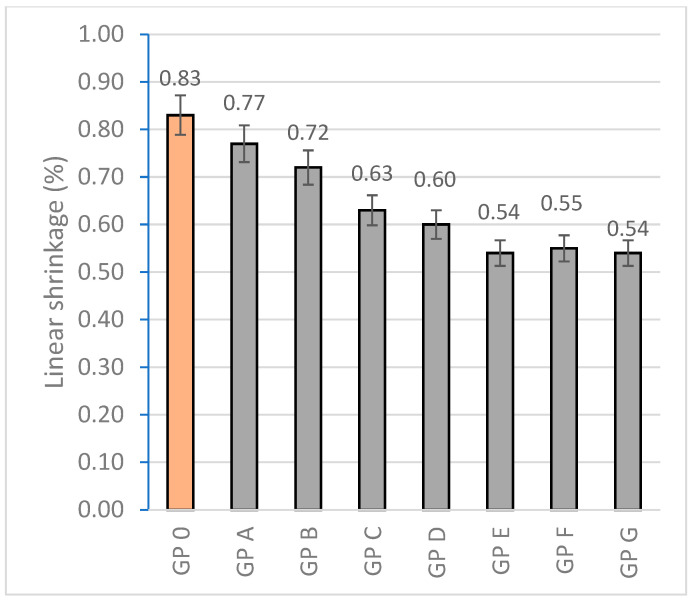
Linear shrinkage (%) of geopolymers.

The linear shrinkage experienced by the specimens is associated with the evaporation of free water and the internal reorganization of the geopolymer gel during the curing process which is related to the increase in densification. All the tested families showed shrinkage values below 1%. The families with varying amounts of OSBA and Na_2_SiO_3_ showed linear shrinkage percentages lower than GP 0 due to the higher specific surface area of the particles related to the spherical morphology of OSBA (Figure 4) which provides volumetric stability and increased reactivity while reducing porosity by favoring granular packing [79]. With the addition of 21% OSBA and the reduction of Na_2_SiO_3_ (28%) (GP G), the linear shrinkage is reduced by approximately 35% compared to GP0. Figure 14 shows the linear shrinkage values for the different families tested.

#### 3.1.3. Determination of Water Absorption by Capillary Action and Immersion

**Figure 15:**
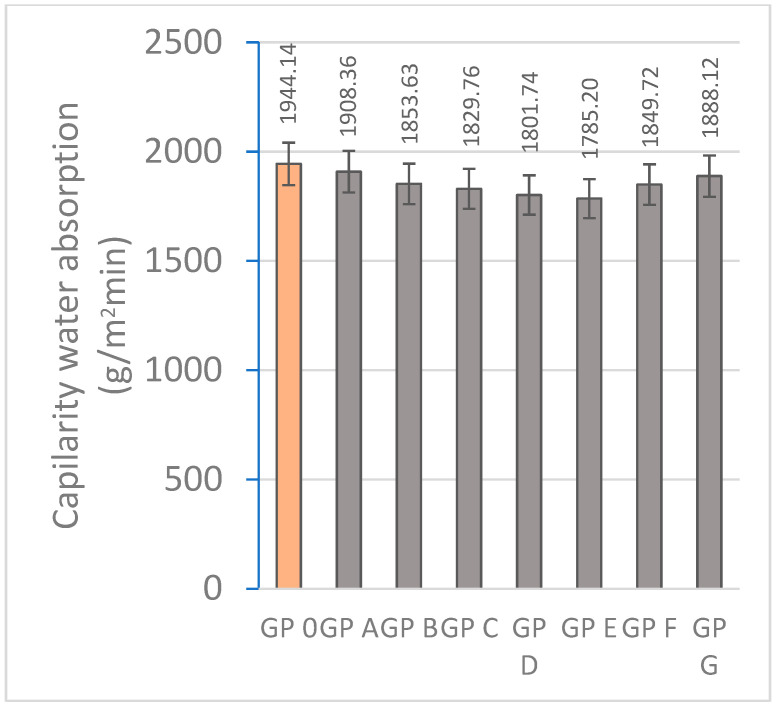
Capillarity water absorption (g/m^2^min) of geopolymers.

**Figure 16:**
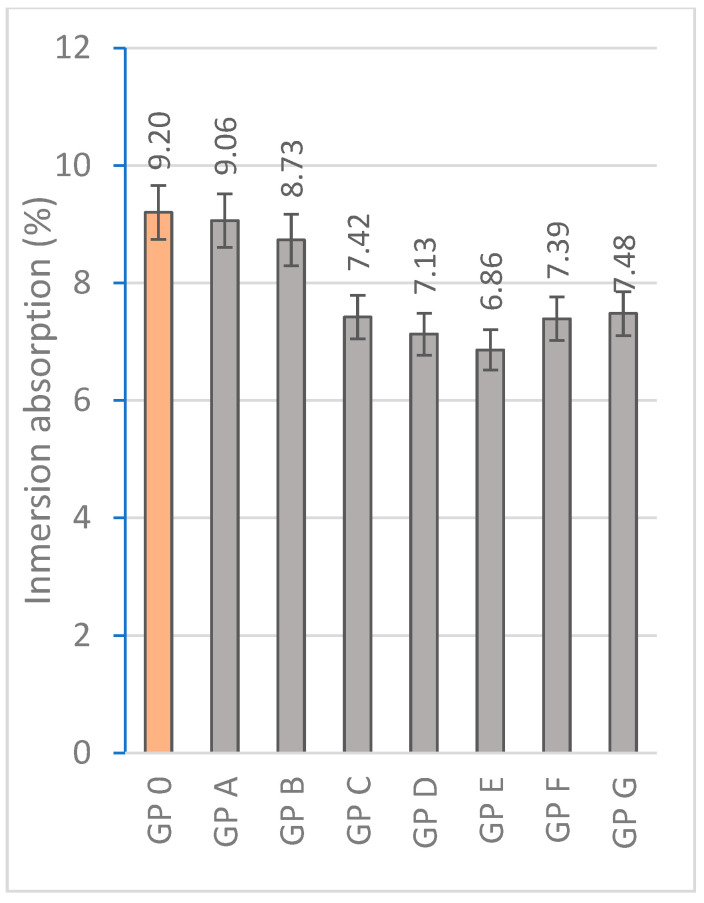
Immersion absorption (%) of geopolymers.

Water absorption is a crucial indicator of material durability and influences performance in extreme environmental conditions. Figure 15 and Figure 16 represent the results of water absorption by capillarity and absorption. The high adsorption of GP-0, GP-A, and GP-B is related to the higher porosity produced by the lower reactivity as these samples do not contain an adequate proportion of alkaline activator and precursor materials. The lower presence of calcium results in less development of the three-dimensional network of geopolymers. In addition, if the hardening reaction does not occur efficiently, a higher porosity is produced, thus reducing the bulk density. The water absorption by immersion of the geopolymers decreases significantly from GP 0 (9.20%) to GP E (6.86%) by 25.43%. The families with a higher proportion of OSBA (GP F and GP G) show higher absorption ratios related to the amount of unreacted OSBA due to the scarcity of diffusion mechanisms due to the lower proportion of Na_2_SiO_3_ in the mixture and the excess of calcium that causes alterations in the formation of the three-dimensional network. Unreacted OSBA particles increase the interaction with water and thus increase the adsorption rate [65].

All the geopolymers showed capillary absorption ratios below 2000 g/m^2^min. Capillary absorption is directly related to the open porosity and the internal structure of the geopolymer which facilitates capillary water transport. OSBA acts as a reactive filler that enhances the geopolymer matrix. GP E shows the best capillary absorption values (1785.20 g/m^2^min) which are related to a better condensation process due to an optimal Na_2_SiO_3_/OSBA ratio (1.0) that favors geopolymerization [80]. The reduced OSBA content in GP A (15%) and GP B (16%) prevents the correct dissolution of Al^3+^ and Si^4+^ ions present in SSCS and CH, producing a more porous matrix.

#### 3.1.4. Determination of Bulk Density

**Figure 17:**
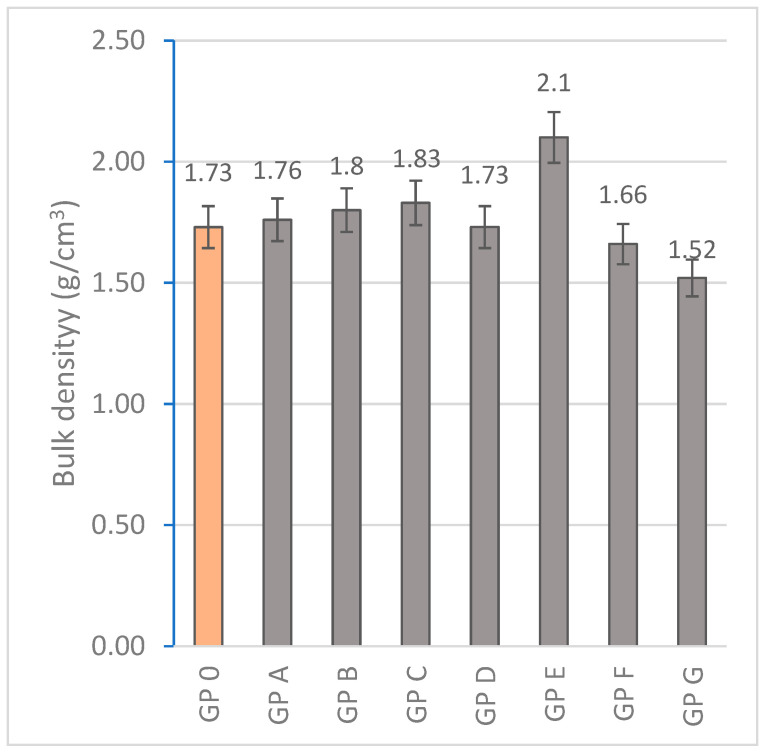
Bulk density (g/cm^3^) of geopolymers.

The densification of the geopolymer matrix occurs during the formation of the reaction products when the aluminosilicate-rich precursors dissolve in the alkaline medium and release silicate (SiO_4_^4−^) and aluminate (AlO_4_^3−^) ions [81]. Increasing the OSBA content from 15% (GP A) to 19% (GP E) resulted in the densification of the matrix (from 1.76 to 2.1 g/cm^3^) related to the higher calcium content. Calcium enhances the dissolution of aluminosilicates in the alkaline medium, which promotes the release of silicate (SiO_4_^4−^) and aluminate (AlO_4_^3−^) ions. Therefore, the hardening reaction is accelerated as the polycondensation of the gel occurs faster and a more compact and less porous structure is generated. However, at OSBA addition ratios of 20% (GP F) and 21% (GP G), there is a decrease in bulk density values (1.66 and 1.52 g/cm^3^, respectively) related to the presence of excess calcium. Excess calcium in the geopolymer matrix can alter crystallization during geopolymer formation and hinder the development of its three-dimensional network structure [82,83]. Figure 17 represents the bulk density results obtained from the experimentation.

**Figure 18:**
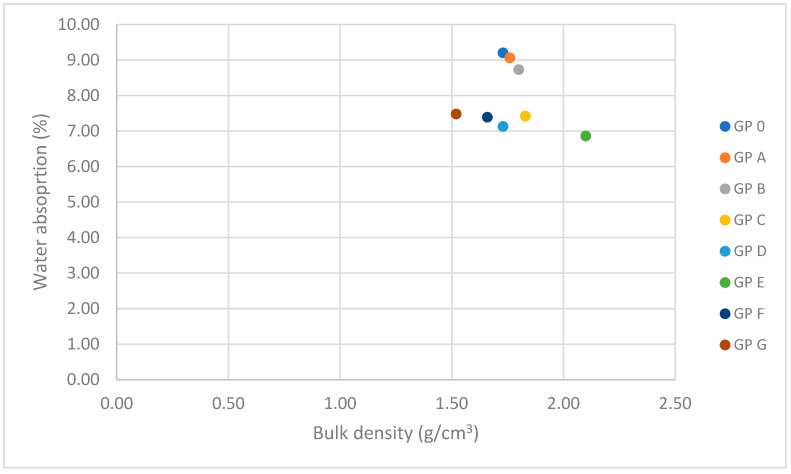
Correlation between water absorption (%) and bulk density (g/cm^3^).

The correlation between water absorption by immersion and bulk density is depicted in Figure 18. It can be seen that as the bulk density of geopolymers increases, there is a decrease in water absorption due to lower porosity and pore connectivity, which hinders water transport and retention [84].

#### 3.1.5. Determination of Porosity

**Figure 19:**
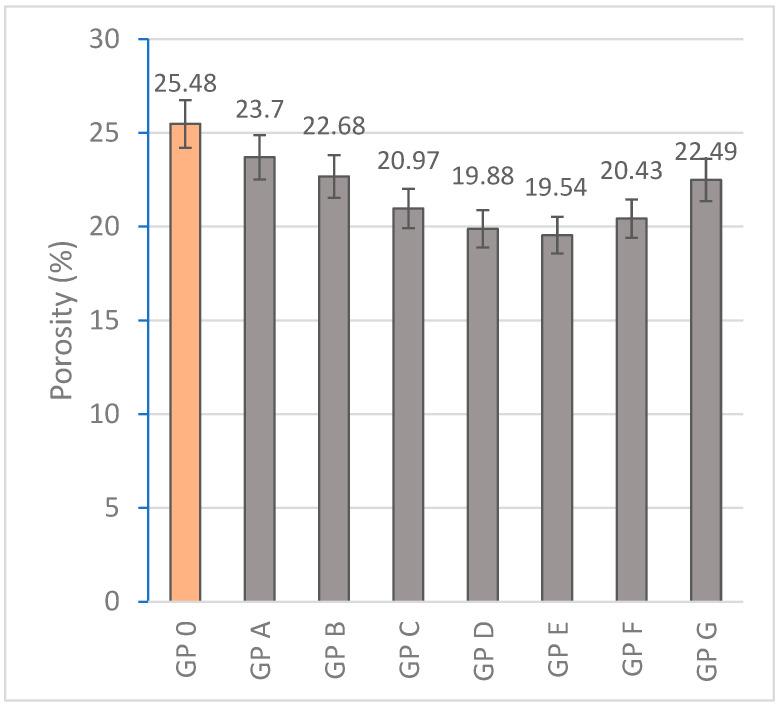
Porosity (%) of geopolymers.

Porosity is directly related to the densification of the geopolymer matrix, so the higher the bulk density, the lower the porosity contained. Na_2_SiO_3_ present in higher proportions produces an increase in the content of soluble silica. The presence in excess produces an incomplete dissolution of the aluminosilicates which increases the porosity [85]. Decreasing the Na_2_SiO_3_/OSBA ratio from GP A to GP E reduces the amount of silica available for N-A-S-H gel formation by decreasing the Si/Al ratio. The increase in the Ca/Si ratio by increasing the amount of OSBA favors the formation of C-A-S-H gel which contributes to the reduction in porosity by filling voids present in the matrix. Similarly, increasing the Na/Si ratio provides more Na^+^ cations that favor the solubility of the aluminosilicates, developing a more uniform microstructure [86]. GP F (20.43%) and GP G (22.49%) show an increase in porosity possibly associated with an excess of calcium and potassium that could be interfering with the formation of a uniform matrix and the development of the three-dimensional network. The porosity in GP 0 (25.48%) is related to the lower densification capacity of the matrix due to a higher content of Na_2_SiO_3_ and NaOH that generate a higher porosity during water evaporation during the curing process [87]. GP E showed the best porosity results (19.54%) associated with an equilibrium Na_2_SiO_3_/OSBA ratio. Figure 19 shows the porosity results obtained from the experimentation.

#### 3.1.6. Determination of Thermal Conductivity

**Figure 20:**
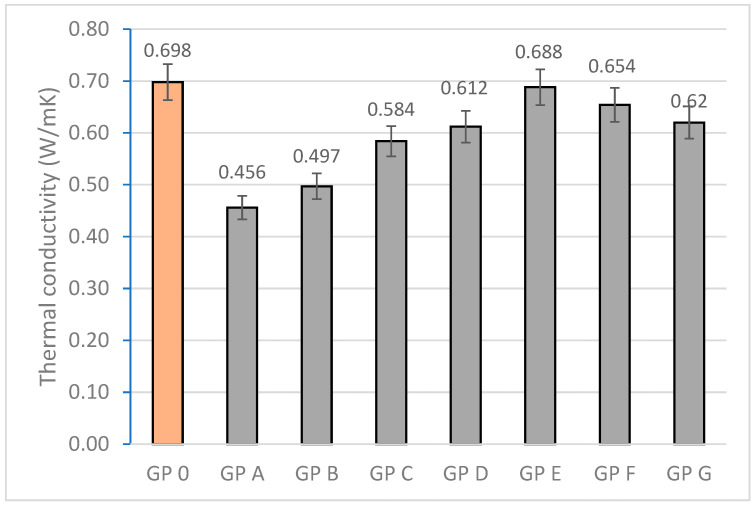
Thermal conductivity (W/mK) of geopolymers.

Thermal conductivity is closely related to the porosity and bulk density parameters. GP D, GP E, and GP F (0.612, 0.688, and 0.654 W/mK) show higher thermal conductivity values due to the higher compaction of the specimens, which facilitates heat transfer by providing a continuous path for heat flow. GP A and GP B (0.456 and 0.497 W/mK) showed the lowest thermal conductivity values related to the higher porosity present in the specimens, which disrupts heat transfer due to the presence of air trapped in the pores. Higher Si/Al ratios favor the formation of a three-dimensional network which improves the structural continuity and, therefore, the thermal conductivity. As discussed above, the increased Ca/Si ratio contributes to the formation of a denser matrix and thus to a more thermally conductive structure. Excess calcium in GP G produces micro defects in the matrix that reduce the conductivity (0.620 W/mK). Figure 20 shows the thermal conductivity values obtained.

#### 3.1.7. Evaluation of Freeze–Thaw Cycles

**Figure 21:**
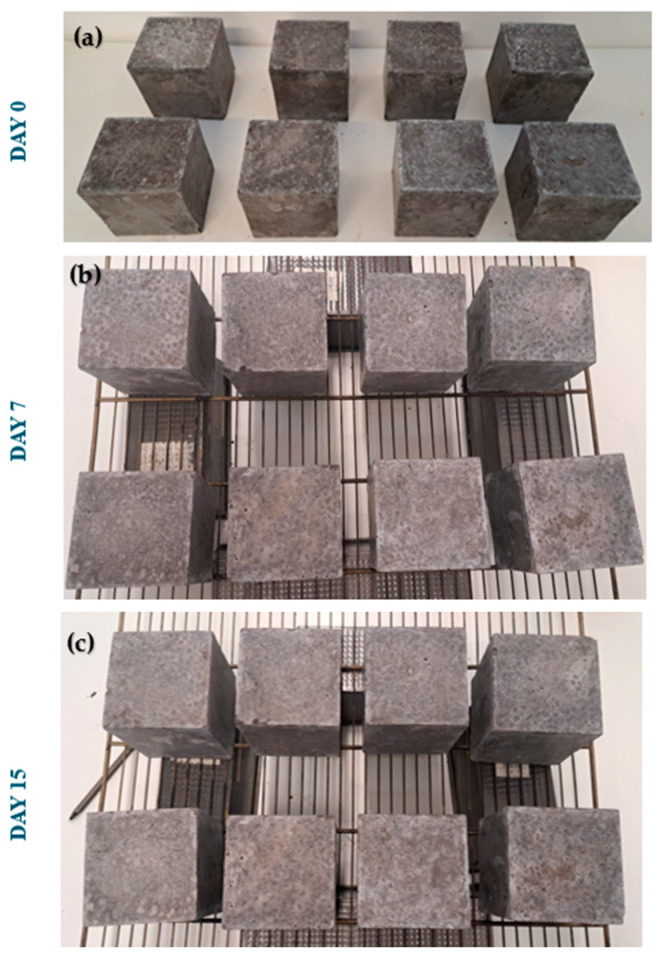
Freeze–thaw resistance at (**a**) 0 days, (**b**) 7 days, and (**c**) 15 days.

Freeze–thaw cycles can cause deterioration of the tested materials through expansion, cracking, or flaking [77,88,89,90]. After the completion of the 15 freeze–thaw cycles and by optical evaluation, it was observed that none of the tested specimens showed cracks or flaking of the surface. Figure 20 shows the test specimens on day 0 of the test (Figure 21a), on day 7 after half of the test time (Figure 21b), and on day 15 after the completion of all the freeze–thaw cycles (Figure 21c). Regarding the colorimetry of the test pieces, it was observed that all of them acquired a whiter shade; however, there were no variations in the texture.

#### 3.1.8. Efflorescence Evaluation

**Figure 22:**
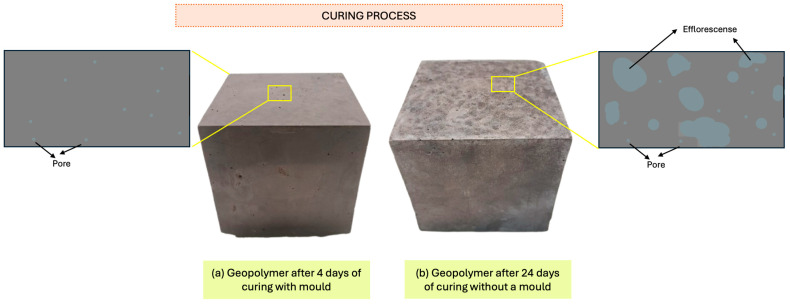
Primary efflorescence of geopolymer after (**a**) 4 days of curing with mold and (**b**) 24 days of curing without mold.

The geopolymers showed the phenomenon of primary efflorescence, a characteristic process that occurs after curing. This phenomenon is observed to varying degrees depending on the conditions to which the geopolymers are exposed during curing. During this process, the surface of the geopolymers becomes whitish in certain areas due to the formation of crystals from the alkaline activator that has not fully reacted with the precursor materials. This phenomenon is clearly visible, as illustrated in Figure 22, which shows the areas affected by this type of efflorescence.

The liquid content in the geopolymer mixture plays a crucial role in the diffusion of water particles (H_2_O) that have not yet fully reacted. These particles tend to move towards the surface of the geopolymer, facilitated by the presence of excess liquid during the curing process. The excess alkaline activator that did not react with the geopolymer components during its formation has the ability to migrate easily to the surface of the material. Once at the surface, this excess activator can come into contact with atmospheric carbon dioxide (CO_2_), leading to a chemical reaction that produces efflorescence. This process is a direct consequence of the interaction between the unreacted components and the environment, resulting in the formation of visible crystals on the surface of the geopolymer [79,91].

However, it is important to note that the formation of secondary efflorescence has not been observed despite the exposure of the geopolymers to adverse environmental conditions, such as low temperatures or prolonged contact with water. This resistance to secondary efflorescence manifests the durability and potential stability of geopolymers in harsh environments, making them an attractive option for applications where weather-resistant building materials are required.

### 3.2. Chemical Characterization of Geopolymers

#### 3.2.1. XRD Analysis

**Figure 23:**
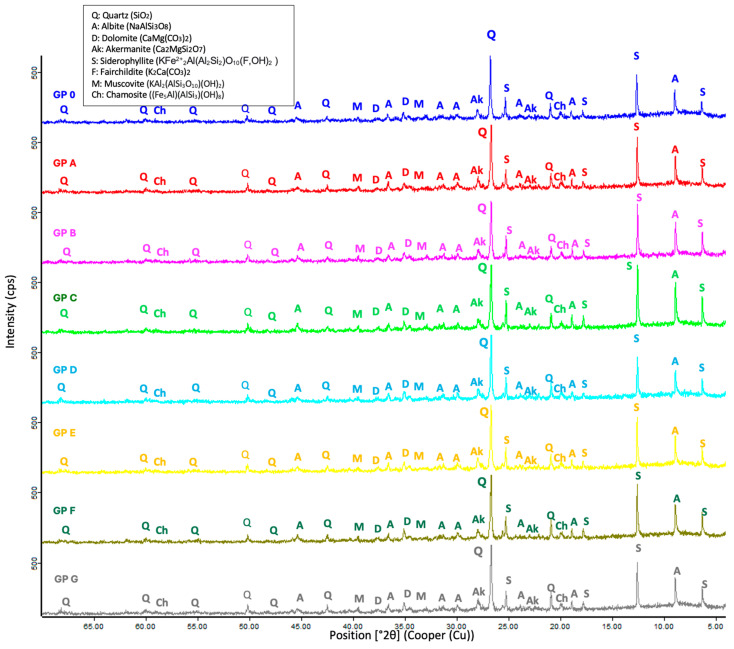
XRD patterns of geopolymers.

The compositional analysis of the geopolymers (Figure 23) shows that the crystalline phases appearing in the aluminosilicate source (SSCS and CH) and in the alkaline activator (OSBA) (Figure 5) appear in the formed geopolymers. Aluminosilicates predominate in the form of quartz (Q) (SiO_2_) and muscovite (M) (KAl_2_(AlSi_3_O_10_)(OH)_2_). The presence of quartz (Q) indicates that the geopolymerization reaction does not occur completely. The intensity of albite (A) (NaAlSi_3_O_8_) decreases with decreasing Na_2_SiO_3_/OSBA ratio, which is related to a higher degree of gel formation [85].

**Figure 24:**
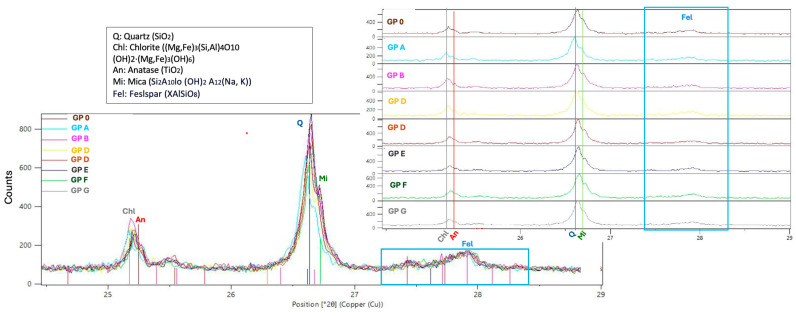
XRD detail between 24° and 29°.

Figure 24 shows a detail of the diffractograms of the specimens between 24° and 29° showing the presence of chlorite (Cl) and mica (Mi) common to metamorphic and sedimentary rocks such as slate [48]; anatase (An) formed during the combustion of OSBA [44]; and quartz (Q) and feldspars (Fel) typical of ceramic materials.

#### 3.2.2. FTIR Analysis

**Figure 25:**
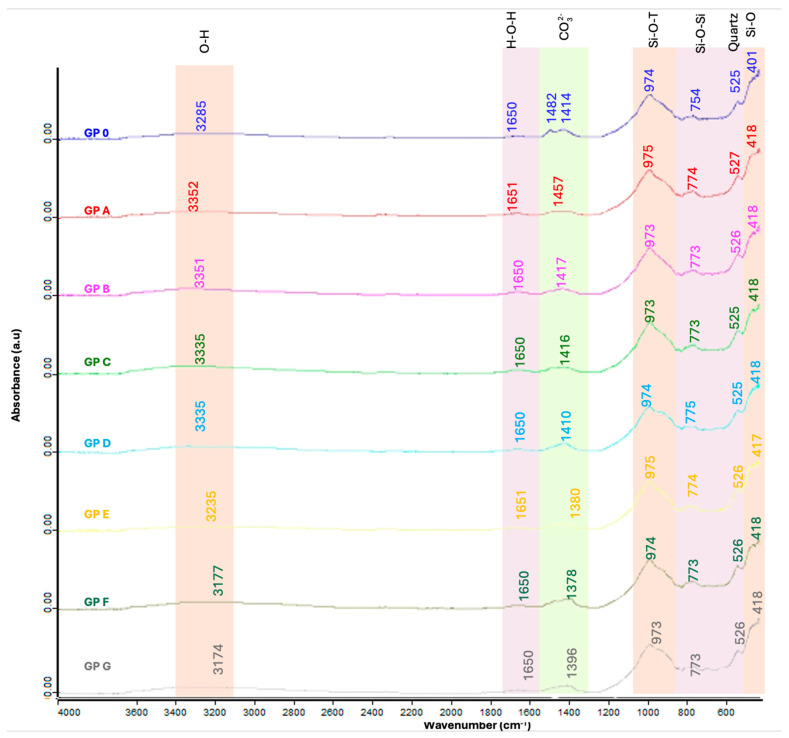
FTIR patterns of geopolymers.

FTIR analysis determines the chemical bands produced during the geopolymerization reaction of the different families formed (Figure 25). It is observed that the amount of OSBA influences the wavenumber with a decrease in the number as the content increases, which is related to a higher densification of the matrix associated with an improvement in the degree of geopolymerization. The bands between 3352 and 3174 cm^−1^ are associated with the stretching vibration of the O-H bond [59,92,93,94]. The bands between 1651 and 1650 cm^−1^ are related to the bending vibration of the H-O-H bond and represent the presence of water in the matrix porosity [54,58]. In the range of 1482 and 1378 cm^−1^ appear the CO asymmetric stretching vibration bands indicating the presence of (CO_3_)^2−^ carbonates [92,94]. The presence of carbonates is related to the decomposition of potassium carbonate into potassium oxide and carbon dioxide during the calcination process of OSBA. The asymmetric Si-O-T stretching vibration between the bands 975–973 cm^−1^ indicates gel formation during the geopolymerization process and is related to the formation of Si-O-Al bonds that provide strength to the matrix as the three-dimensional networks characteristic of geopolymers are formed [54,91,93]. The bands between 774 and 754 cm^−1^ are associated with the bending symmetric stretching vibration of the Si-O-Si bonds in the unreacted silica network [54]. Between 527 and 526 cm^−1^, quartz bands appear, indicating the presence of unreacted quartz [92], while between 418 and 401 cm^−1^, Si-O bands appear [58,94]. Table 9 shows the characteristic peaks of the FTIR spectra of the studied geopolymer families.

### 3.3. Mechanical Characterization of Geopolymers

The mechanical strength of the geopolymers was evaluated at 7, 14, and 28 days of curing. The curing conditions of the geopolymers were kept constant for all the geopolymer families manufactured; therefore, the obtained values of compressive strength are influenced by the reactivity of the blends, the molar ratios of the majority elements, and phenomena such as efflorescence [79]. The presence of Na_2_SiO_3_ in the alkaline activator provides soluble silicon that contributes to the increase in the Si/Al ratio while producing an increase in the degree of condensation of the geopolymerization reaction. A high percentage of Na_2_SiO_3_ can lead to an unreacted excess which decreases the potential of the geopolymerization reaction and increases the viscosity of the geopolymer mortar. The increase in matrix viscosity is related to decreasing pH values [86].

**Figure 26:**
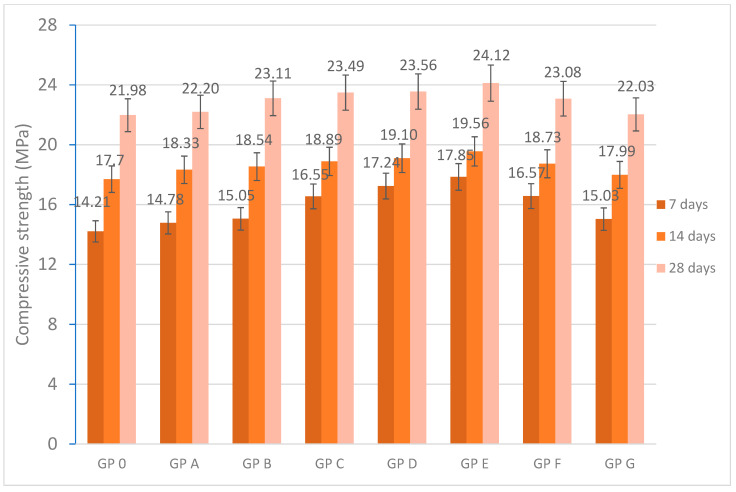
Compressive strength (MPa) of geopolymer.

Figure 26 shows the compressive strength values obtained at 7, 14, and 28 days of curing. As can be seen, the curing time influences the strength values with an increase in the values from 7 to 28 days of curing. The increase is magnified during the curing period from 14 to 28 days which is related to the repolymerization reaction produced during gel formation from alkaline activation [95]. The influence of OSBA on the alkaline activator was visible from 15 (GP A) to 19% (GP E) by 8.41% from 22.20 to 23.56 MPa.

GP C, GP D, and GP E showed the best compressive strength values, which is related to a better Na_2_SiO_3_ ratio. As shown above, the three families of specimens presented more densified matrices which are related to a better mechanism in the geopolymerization reaction [96]. The decrease in the Si/Al and Ca/Si molar ratios (Table 6) with the decrease in the Na_2_SiO_3_/OSBA ratio (Table 5) produces the equilibrium of the characteristic three-dimensional networks that form the geopolymers. The justification lies in the increase in the number of Si-O-Si and Si-O-Al bonds [97]. On the other hand, the excess of OSBA in the alkaline activator can produce imbalances in the three-dimensional network caused by high concentrations of Ca^2+^, Si^4+^, and Al^3+^ leading to a decrease in the rate of the reaction mechanism. On the contrary, GP A and GP B showed lower resistance values related to the lower OSBA content which produces a lower degree of densification by decreasing the reactivity of the alkaline activator with decreasing K/Si and Na/Si ratios (Table 6).

Regarding the Si/Al ratio, opinions differ. Some authors highlight improvements in compressive strength values with values between 3 and 5 [81], while others highlight that mechanical properties improve with a ratio between 1.5 and 2.5 [35,98,99].

The calcium present in OSBA influences the Ca/Si molar ratio, improving gel stability when present in moderate proportions. High concentrations lead to a decrease in the reaction mechanism and to the formation of calcium hydroxide (Ca(OH)_2_) whose presence causes the appearance of carbonates while decreasing the number of Si-O-Al bonds [100]. The decrease in compressive strength values in GP F (23.08 MPa) and GP G (22.03 MPa) is related to the low reactivity of the alkaline activator produced by an excess of OSBA.

**Figure 27:**
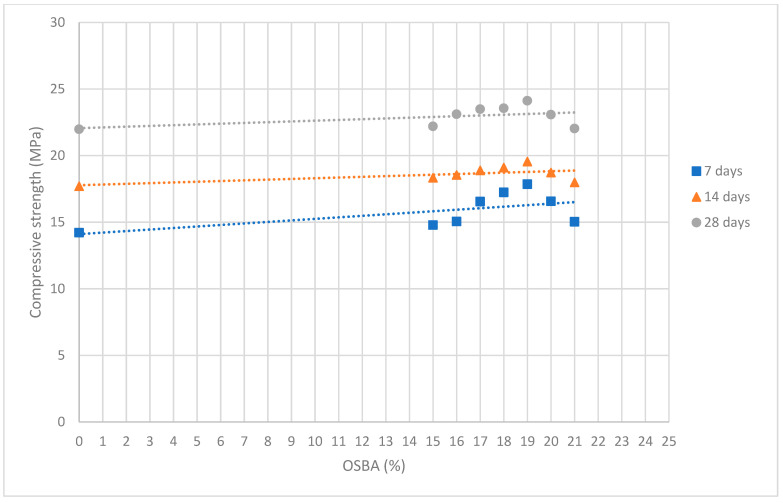
Relationship between the percentage of OSBA added and the compressive strength values.

Figure 26 shows the compressive strength values obtained at 7, 14, and 28 days graphically. Figure 27 shows the relationship between the percentage of OSBA added and the compressive strength values.

### 3.4. Microscopic Characterization of Geopolymers

**Figure 28:**
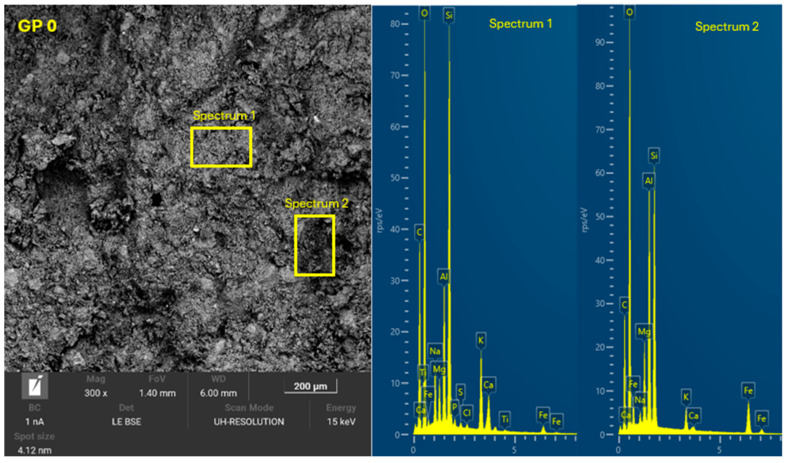
SEM of GP 0: entire area of SEM secondary 300× image and SEM retro-dispersed with selection of 2 areas of spectra for EDX analysis.

**Figure 29:**
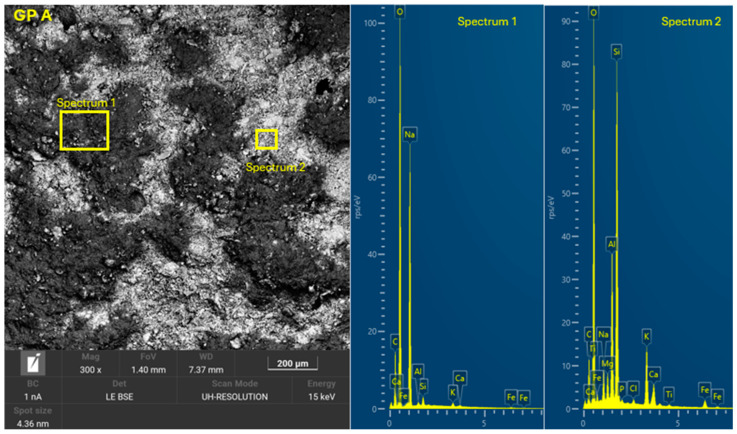
SEM of GP A: entire area of SEM secondary 300× image and SEM retro-dispersed with selection of 2 areas of spectra for EDX analysis.

**Figure 30:**
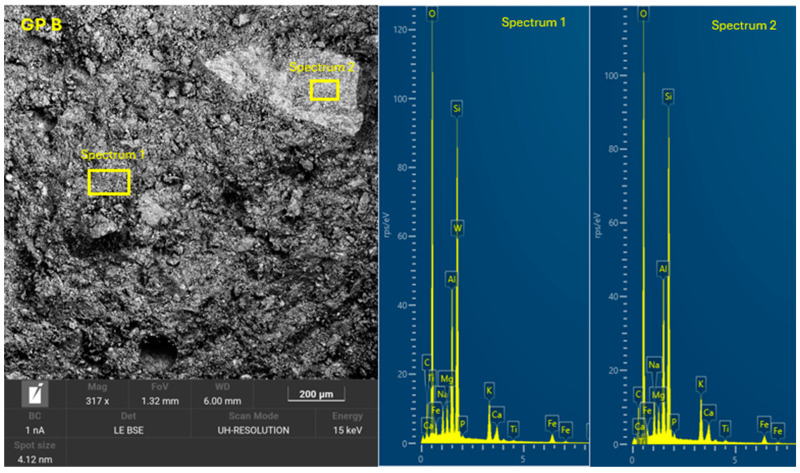
SEM of GP B: entire area of SEM secondary 300× image and SEM retro-dispersed with selection of 2 areas of spectra for EDX analysis.

**Figure 31:**
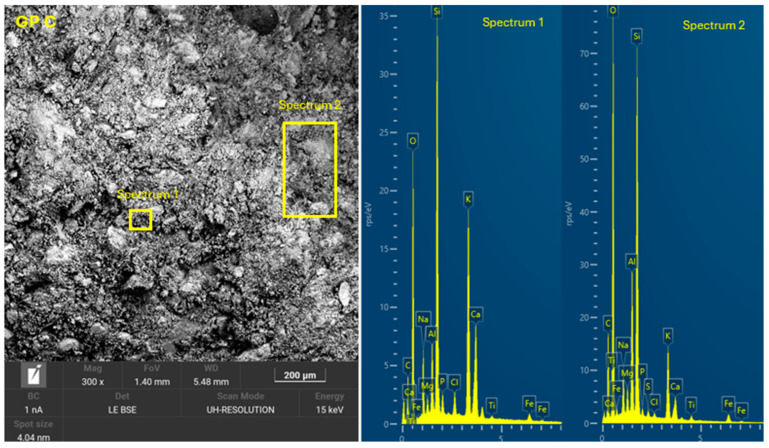
SEM of GP C: entire area of SEM secondary 300× image and SEM retro-dispersed with selection of 2 areas of spectra for EDX analysis.

**Figure 32:**
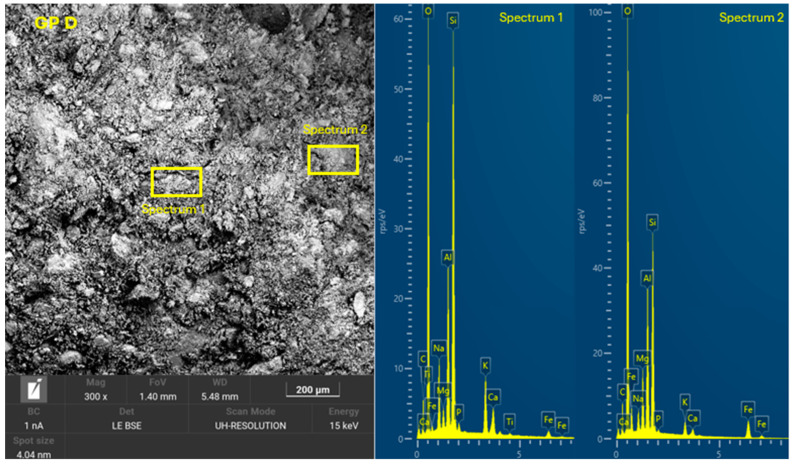
SEM of GP D: entire area of SEM secondary 300× image and SEM retro-dispersed with selection of 2 areas of spectra for EDX analysis.

**Figure 33:**
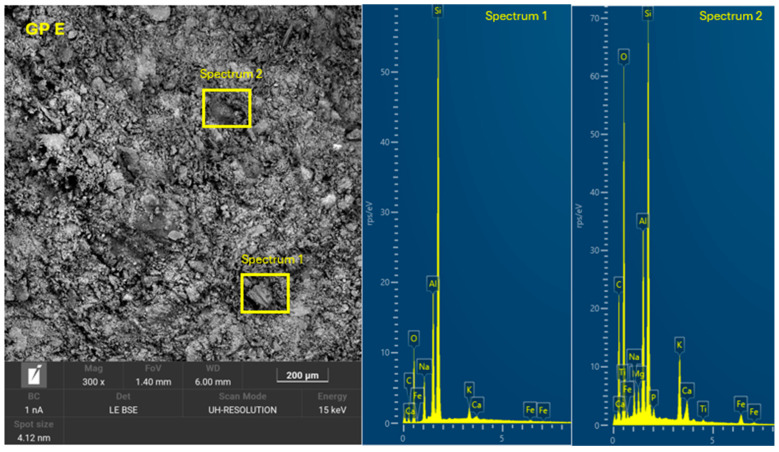
SEM of GP E: entire area of SEM secondary 300× image and SEM retro-dispersed with selection of 2 areas of spectra for EDX analysis.

**Figure 34:**
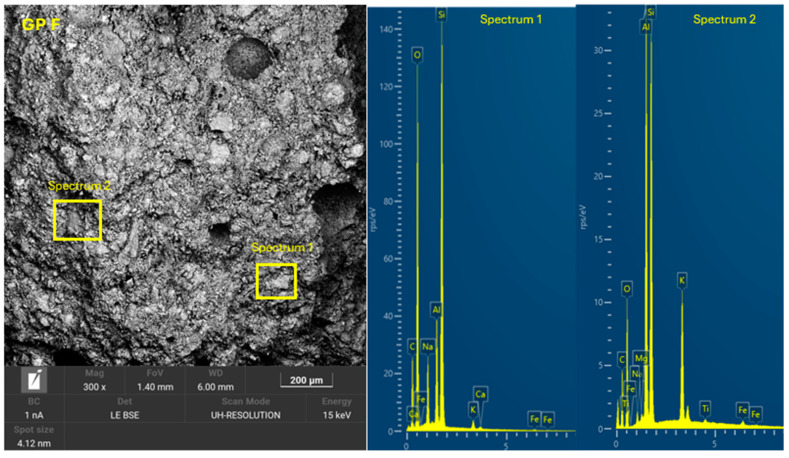
SEM of GP F: entire area of SEM secondary 300× image and SEM retro-dispersed with selection of 2 areas of spectra for EDX analysis.

**Figure 35:**
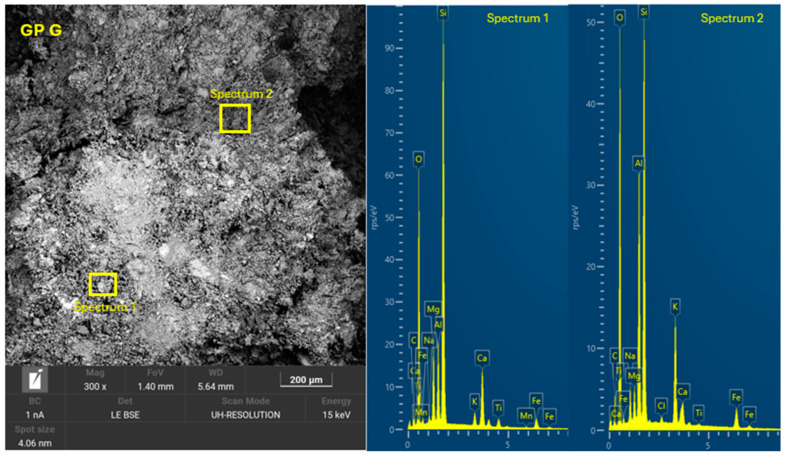
SEM of GP G: entire area of SEM secondary 300× image and SEM retro-dispersed with selection of 2 areas of spectra for EDX analysis.

The 8 geopolymer families were analyzed by SEM-EDX (Microscope Carl Zeiss Merlin, Zeiss GmbH, Jena, Germany) after 28 days of curing (Figure 28, Figure 29, Figure 30, Figure 31, Figure 32, Figure 33, Figure 34 and Figure 35). The EDX spectra revealed that the hydration products were mainly composed of calcium (Ca), sodium (Na), silicon (Si), aluminum (Al), and potassium (K) species. Hydration products (C-A-S-H and N-A-S-H gel) occur in all families as confirmed by EDS analysis. The presence of both was confirmed by the occurrence of Ca (C-A-S-H gel) and Na (N-A-S-H gel). In GP F and GP G, a less compact microstructure was observed, showing that an excess of OSBA slows down the geopolymerization reaction and gel formation, leading to a decrease in compressive strength [83]. The presence of magnesium (Mg) is associated with the presence of clay minerals present in CH [101]. Considering the XRD results, most of the crystals must be quartz (Q) and feldspars (Fel). GP C, GP D, and GP E showed smaller crystalline phases with a denser structure related to the decrease in Si/Al and Ca/Si molar ratios. The lower presence of C-A-S-H gel in GP E is related to the better balance of Si/Al and Na/Si molar ratios providing OH^−^ and Na^+^ ions, leading to better Si-O-Si, Al-O-Si, and Ca-O bond cleavage [102]. GP A shows a heterogeneous matrix composed of unreacted Na_2_SiO_3_ due to an excess in the Na_2_SiO_3_/OSBA ratio.

## 4. Conclusions

This study evaluated the feasibility of using OSBA for the activation of geopolymers from SSCS and CH on physical, chemical, mechanical, and microstructural properties. The main findings are described below:

- Physical characterization showed that GP E performed best with a Na_2_SiO_3_/OSBA ratio of 1.0 which favors the formation of (C, N)-A-S-H gels and forms dense matrices with reduced porosity.
- The XRD analysis of the mortars indicates that the geopolymers are composed of crystalline phases with quartz inclusions, suggesting the incomplete dissolution of the precursors. The presence of albite plays a key role in the formation of the geopolymer gel, influencing the structure and cohesion of the matrix, which may affect both the reactivity of the system and its mechanical properties.
- The compressive strength results demonstrate the feasibility of using OSBA in the activation of SSCS- and CH-based geopolymers. GP E showed an increase in compressive strength value and reached 24.12 MPa at 28 days of curing, 15.16% higher than GP 0 influenced by the reactivity of the geopolymer gel.
- SEM-EDX showed the formation of C-A-S-H and N-A-S-H phases present in the geopolymer gel forming an interconnected network. The resulting gel composition is characterized by balanced Si/Al and Na/Si ratios.
- The manufacturing process of geopolymers would be scalable due to the fact that high curing temperatures are not required and that the main materials come from the by-products of other industries that are obtained at low cost.

Based on these findings, the use of SSCS and CH waste from the mining and construction industry in the manufacture of OSBA-activated geopolymers from the thermal industry represents an effective route for the valorization of waste from different industries.

## Figures and Tables

**Table 1:** Element analysis of SSCS, OSBA, and CH.

Raw Material	SSCS	OSBA	CH
C (%)	0.580	3.870	0.424
N (%)	0.030	0.011	0.001
H (%)	0.380	3.035	0.001

**Table 2:** Density of SSCS, OSBA, and CH.

Raw Materials	Density (Kg/m^3^)
SSCS	2.49 ± 0.15
OSBA	2.74 ± 0.19
CH	2.53 ± 0.21

**Table 3:** Chemical components of SSCS, OSBA, and CH.

Raw Material	SSCS	OSBA	CH
K_2_O	4.54	27.48	5.12
CaO	0.462	34.22	7.22
Al_2_O_3_	21.97	0.779	15.41
SiO_2_	50.34	4.12	58.98
P_2_O_5_	0.263	1.85	0.155
MgO	2.50	3.82	2.40
Fe_2_O_3_	10.38	0.94	7.84
Cl	-	0.092	0.028
Na_2_O	1.37	0.761	0.367
SO_3_	0.501	0.361	0.863
TiO_2_	1.22	0.060	0.882
MnO	0.099	0.125	0.109
V_2_O_5_	0.035	-	0.026
CuO	0.027	0.055	0.011
Cr_2_O_3_	0.021	0.048	0.017
ZnO	0.016	-	0.046
NiO	-	0.041	-

**Table 4:** Characteristic absorption peaks of FTIR of SSCS, OSBA, and CH.

Function Group	Wavenumber Range (cm^−1^)	FTIR Peaks (cm^−1^)			References
Raw Materials		SSCS	OSBA	CH	
Stretching vibration O-H	3626–3002	3326, 3381	3002	-	[53]
Bending vibration H-O-H	1648	-	1648	-	[53,54]
Asymmetric stretching vibration CO	1417	-	1417	-	[55]
Asymmetric stretching vibration Si-O-T	972–921	971, 921	-	972	[56,57]
CO bonds vibrations in carbonate groups	879–827	827	879	-	[58,59]
Bending symmetric stretching Si-O-Si	794–749	794, 779, 749	-	775	[58,59,60]
Bending vibration in quartz	693–629	693, 629	662	678	[60]
Bending vibration Si-O	525–418	525	511, 432	432, 418	[61,62]

**Table 5:** Mix proportion of geopolymers containing SSCS, CH, OSBA, NaOH, and Na_2_SiO_3_.

Specimen	SSCS (%)	CH (%)	OSBA (%)	Na_2_SiO_3_ (%)	W_d_ (%)	NaOH 12M (%)	Na_2_SiO_3_/OSBA	NaOH/OSBA	Liquid/Binder Ratio	pH Alkaline Solution
GP 0	35.0	0.0	0.0	37.0	11.0	17	-	1.32	1.86	12.14
GP A	25.0	15.0	15.0	34.0	11.0	0	1.31	-	1.50	13.41
GP B	25.0	15.0	16.0	32.0	12.0	0	1.22	-	1.50	13.28
GP C	25.0	15.0	17.0	30.0	13.0	0	1.14	-	1.50	13.19
GP D	25.0	15.0	18.0	28.0	14.0	0	1.07	-	1.50	13.01
GP E	25.0	15.0	19.0	26.0	15.0	0	1.00	-	1.50	12.92
GP F	25.0	15.0	20.0	24.0	16.0	0	0.94	-	1.50	12.87
GP G	25.0	15.0	21.0	22.0	17.0	0	0.88	-	1.50	12.75

**Table 6:** Molar ratio Si/Al, Ca/Si, K/Si, and Na/Si of geopolymers.

Specimen	Si/Al	Ca/Si	K/Si	Na/Si
GP 0	4.65	0.01	0.49	0.07
GP A	4.92	0.21	1.00	0.19
GP B	4.79	0.26	1.04	0.21
GP C	4.66	0.27	1.08	0.22
GP D	4.54	0.31	1.13	0.23
GP E	4.41	0.34	1.18	0.25
GP F	4.29	0.37	1.24	0.26
GP G	4.16	0.40	1.31	0.28

**Table 7:** Parameter, standard, and equipment used.

Parameter	Standard	Equipment
Weight loss	-	RB-30KG Cobos (Balanzas Cobos, Hospitalet de Llobregat, Spain)
Linear shrinkage	UNE-EN 772-16 [68]	Digital gauge
Capillary water absorption	UNE-EN 772-11 [69]	Stopwatch and balance RB-30KG Cobos
Cold water absorption	UNE-EN 772-21 [70]	Thermostatic bath Bunsen and balance RB-30KG Cobos (Bunsen, Madrid, Spain)
Boiling water absorption	UNE-EN 772-7 [71]	Thermostatic bath Bunsen and balance RB-30KG Cobos
Bulk density and open porosity	UNE-EN 772-4 [72]	Hydrostatic balance
Freeze–thaw resistance	UNE-EN 15304 [73]	Freezer
Thermal conductivity	UNE-EN 12667:2002 [74]	HFM 446 Lambda Eco-Line Netzsch (Netzsch, High Franconia, Germany)
Compressive strength	UNE-EN 772-1:2001+A1:2016 [75]	Shimadzu AG-300 KNX (Shimadzu, Korneuburg, Austria)
DRX	-	Pioner S4 Explorer Bruker (Bruker AXS GmbH, Karlsruhe, Germany)
FTIR	-	FT-IR Vertex 70 Bruker (Bruker AXS GmbH, Karlsruhe, Germany)
SEM-EDX	-	Microscope Carl Zeiss Merlin (Zeiss GmbH, Jena, Germany)

**Table 8:** Results of physical parameters of geopolymers.

Specimen	Mass Loss (%)	Linear Shrinkage (%)	Capillarity Water Absorption (g/m^2^min)	Immersion Absorption (%)	Bulk Density (g/cm^3^)	Porosity (%)	Thermal Conductivity (W/mK)
GP 0	4.05 ± 0.41	0.83 ± 0.10	1944.14 ± 68	9.20 ± 0.18	1.73 ± 0.15	25.48 ± 2.06	0.698 ± 0.015
GP A	3.89 ± 0.35	0.77 ± 0.14	1908.36 ± 71	9.06 ± 0.15	1.76 ± 0.08	23.70 ± 4.38	0.456 ± 0.087
GP B	3.83 ± 0.46	0.72 ± 0.11	1852.63 ± 48	8.73 ± 0.20	1.80 ± 0.21	22.68 ± 3.80	0.497 ± 0.041
GP C	3.70 ± 0.37	0.62 ± 0.09	1801.74 ± 56	7.42 ± 0.13	1.83 ± 0.23	20.97 ± 1.44	0.584 ± 0.093
GP D	3.59 ± 0.29	0.60 ± 0.12	1829.76 ± 32	7.13 ± 0.09	2.10 ± 0.19	19.54 ± 2.31	0.612 ± 0.105
GP E	3.55 ± 0.30	0.54 ± 0.13	1785.20 ± 41	6.86 ± 0.22	1.73 ± 0.17	19.88 ± 2.47	0.688 ± 0.056
GP F	3.61 ± 0.45	0.55 ± 0.09	1849.72 ± 44	7.39 ± 0.15	1.66 ± 0.21	20.43 ± 2.27	0.654 ± 0.09
GP G	3.61 ± 0.32	0.54 ± 0.11	1888.12 ± 28	7.48 ± 0.14	1.52 ± 0.15	22.49 ± 3.71	0.620 ± 0.110

**Table 9:** Characteristic absorption peaks of FTIR spectra.

Function Group	Wavenumber Range (cm^−1^)	FTIR Peaks (cm^−1^)								References
Raw Materials		GP 0	GP A	GP B	GP C	GP D	GP E	GP F	GP G	
Stretching vibration O-H	3352–3174	3285	3352	3351	3335	3335	3235	3177	3174	[59,92,93,94]
Bending vibration H-O-H	1651–1650	1650	1651	1650	1650	1650	1651	1650	1650	[54,58]
Asymmetric stretching vibration CO	1482–1378	1482	1457	1417	1416	1410	1380	1378	1396	[92,94]
Asymmetric stretching vibration Si-O-T	975–973	974	975	973	973	974	975	974	973	[54,91,93]
Bending symmetric stretching Si-O-Si	774–754	754	774	773	773	775	774	773	773	[54]
Bending vibration in quartz	527–526	525	527	526	525	525	526	526	526	[92]
Bending vibration Si-O	418–401	401	418	418	418	418	418	418	418	[58,94]

## Data Availability

Data are contained within the article.
